# Effects of nutrition intervention strategies in the primary prevention of overweight and obesity in school settings: a protocol for a systematic review and network meta-analysis

**DOI:** 10.1186/s13643-021-01661-1

**Published:** 2021-04-22

**Authors:** Edris Nury, Jakub Morze, Kathrin Grummich, Gerta Rücker, Georg Hoffmann, Claudia M. Angele, Jürgen M. Steinacker, Johanna Conrad, Daniela Schmid, Jörg J. Meerpohl, Lukas Schwingshackl

**Affiliations:** 1 Institute for Evidence in Medicine, Medical Center - University of Freiburg, Faculty of Medicine, University of Freiburg, Freiburg, Germany; 2 Department of Cardiology and Internal Diseases, University of Warmia and Mazury, Olsztyn, Poland; 3 Department of Human Nutrition, University of Warmia and Mazury, Olsztyn, Poland; 4 Cochrane Germany Foundation, Cochrane Germany, Freiburg, Germany; 5 Institute of Medical Biometry and Statistics, Medical Center - University of Freiburg, Faculty of Medicine, University of Freiburg, Freiburg, Germany; 6 Department of Nutritional Sciences, University of Vienna, Vienna, Austria; 7 Centre for Teacher Education, University of Vienna, Vienna, Austria; 8 Division of Sports and Rehabilitation Medicine, Ulm University Hospital, Ulm, Germany; 9 Science Department, German Nutrition Society, Bonn, Germany; 10 Division for Quantitative Methods in Public Health and Health Services Research, Department of Public Health, Health Services Research and Health Technology Assessment, UMIT - Private University for Health Sciences, Medical Informatics and Technology, Hall in Tyrol, Austria

**Keywords:** Nutrition interventions, School setting, Obesity, Primary prevention, Network meta-analysis, Systematic review

## Abstract

**Background:**

Overweight and obesity in children and adolescents are major public health challenges affecting quality of life and representing important risk factors in the development of non-communicable diseases. School environments provide great possibilities for the primary prevention of overweight and obesity and different school-based nutrition interventions are available. However, existing research on school-based nutrition interventions has important limitations and no network meta-analysis (NMA) has been performed yet to compare all available interventions. Therefore, the present research project aims to investigate the impact of different nutrition interventions in the school setting by comparing and ranking them using NMA methodology.

**Methods/design:**

A systematic literature search will be performed in 11 electronic databases (PubMed, the Cochrane Library, Web of Science, ERIC, PsycINFO, CAB Abstracts, Campbell Library, BiblioMap EPPI, Australian Education Index, Joanna Briggs Institute Evidence-Based Practice Database and Practice-based Evidence in Nutrition Database). Parallel or cluster randomized controlled trials (RCTs) meeting the following criteria will be included: (1) generally healthy school students aged 4–18 years, (2) school-based intervention with ≥ 1 nutrition component, and (3) assessed anthropometric (overweight/obesity risk, body weight change, weight *Z*-score, [standardized] body mass index, body fat, waist circumference) and/or diet-quality measures (daily intake of fruits and vegetables, fat, and sugar-sweetened beverages). Random effects pairwise and NMA will be performed for these outcomes and surface under the cumulative ranking curve (SUCRA) estimated (*P*-score). Where possible, component NMA (CNMA) will be used additionally. Subgroup analyses are carried out for intervention duration, gender, age of school students, socioeconomic status, and geographical location, and sensitivity analyses by excluding high risk of bias RCTs.

**Discussion:**

This systematic review and NMA will be the first to both directly and indirectly compare and rank different school-based nutrition interventions for the primary prevention of overweight and obesity in childhood and adolescence. Our analyses will provide important insights about the effects of the different interventions and show which are the most promising. The results of our study can help inform the design of new studies and will be of value to anyone interested in developing successful, evidence-based nutrition interventions in school settings.

**Systematic review registration:**

PROSPERO: CRD42020220451.

**Supplementary Information:**

The online version contains supplementary material available at 10.1186/s13643-021-01661-1.

## Background

The primary prevention of overweight and obesity in children and adolescents is a public health priority. Compared to normal-weight peers, children with overweight and obesity more often suffer from higher blood pressure and metabolic disorders such as impaired glucose metabolism, insulin resistance, and dyslipidemia, all important risk factors for cardiovascular diseases (CVD) [1], the leading cause of death worldwide [2]. Since increased body weight is considered socially undesirable, this is often accompanied by low self-esteem resulting in depression or eating disorders, thereby perpetuating these health-related disorders [3]. Thus, a consistent association has been observed between an increased body mass index (BMI) during childhood and adolescence and an increased risk of hypertension, type 2 diabetes, and CVD in adulthood [4]. Recent global estimates are alarming and show that approximately 38 million (6%) children under 5 years of age were overweight in 2019 [5, 6], while in the year 2016 nearly 340 million (18%) children and adolescents aged 5–19 years were either overweight or obese [7, 8]. The main cause for the development of overweight and obesity is a sustained imbalance between energy intake and energy expenditure [9]. A suboptimal diet, sedentary behavior, and physical inactivity are among the most important influencing factors for increased body weight [10]. In particular, low consumption of fruit and vegetables [11] and high consumption of sugar-sweetened beverages (SSB) [12–16] and dietary sugars [17] are considered dietary risk factors for obesity in childhood and adolescence [18]. It has been well established that children and adolescents in most countries and regions of the world do not meet World Health Organization (WHO) intake recommendations for fruits and vegetable [19–22], i.e., 400 g or 5 servings of 80 g daily [21]. Recommendations by the WHO stating that the daily intake of free sugars should be less than 10% and preferably less than 5% of total energy intake (TEI) [23] are also not met by children and adolescents in most world regions [24, 25]. In addition, research has shown that children’s intake of added sugars increases starting from the age of 1 year, with intake numbers being highest in school-aged children and adolescents (up to 19% TEI) [24]. For SSB in particular, several studies have shown that they contribute significantly (range 6–15%) to the daily TEI of children and adolescents of school age, nearing or even exceeding the WHO recommendations for total free sugar intake [26–30]. Children’s and adolescents’ understanding of health and their health behaviors are strongly influenced by various environmental factors [31], necessitating that their living environments be made more health-oriented. An important living environment where children and adolescents spend much time, including intake of meals, is school. The school setting therefore can offer great opportunities for health promotion and primary prevention. Several systematic reviews and pairwise meta-analyses have investigated the effects of nutrition interventions (e.g., nutrition-friendly school initiatives) in school settings [32–42]. However, these publications did not consider overweight or obesity as an outcome [32, 33], included randomized controlled trials (RCTs) without a nutrition component [36, 37], did not perform meta-analysis (MA) [34], were limited to the treatment of overweight and obesity [41, 42], or compared only one specific nutrition intervention (e.g., nutrition education and training) with a control group [40].

In sum, to date, no network meta-analysis (NMA) on the effects of different nutrition interventions in the school setting for the primary prevention of overweight and obesity is available. While the aforementioned MAs have chosen the traditional approach of paired MA to compare two interventions (e.g., nutrition education vs. control), the innovative approach of the present research focuses on the methodological aspects and benefits of NMA. As usual, a variety of different types of school-based prevention strategies are available, it is necessary to compare all strategies. This is where NMA has two major advantages over pairwise MA: (1) it allows simultaneous comparison of more than two strategies, even when direct comparisons are missing, i.e., by using indirect comparisons, and (2) it also allows ranking from “best” to “worst” in order to create hierarchies, e.g., concerning the effects of overweight and obesity prevention strategies.

Therefore, the aim of the present research project is to investigate the impact of different nutrition interventions in the school setting, combine the direct and indirect evidence, rank the different nutrition interventions for effects on anthropometric outcomes (overweight or obesity risk, body weight change, weight *Z*-score, BMI or standardized BMI (zBMI), body fat, and waist circumference), and measure diet quality using NMA methodology.

## Methods/design

The proposed systematic review was registered in PROSPERO International Prospective Register of Systematic Reviews (registration number: CRD42020220451). The current study protocol has been designed, conducted, and reported in compliance with quality standards (Preferred Reporting Items for Systematic Reviews and Meta-Analyses [PRISMA]) for reporting systematic review and NMA protocols [43] (additional file 1).

### Eligibility criteria

RCTs meeting all of the following criteria will be considered and included in the NMA:

#### Types of participants and setting

Generally healthy children and adolescents between the ages of 4 and 18 years who attend schools, including primary schools, secondary school, and schools for children with special educational needs will be included. The definition of the target group (including age groups) and setting was based on a highly cited Cochrane review [35] that investigated the WHO Health Promoting School (HPS) framework for improving the health, well-being, and performance at school. Whereas the Cochrane review focused on a variety of different health interventions (e.g., physical activity, nutrition, mental health, etc.) and only included nutrition interventions that conformed to the HPS approach (i.e., whole-school health promotion through healthy school policies, physical school environments, social school environments, individual health skills and action competencies, community links, and health services [44]), the present research project considers all nutrition interventions in the school setting. Studies that include pre-school children in addition to school-aged children will be excluded, when study results are not separately reported for the school-aged children. Similarly, RCTs that primarily include both overweight and obese children/adolescents without presenting results separately for the two groups will also be excluded. Moreover, RCTs with a study population of obese children only will also not be considered, as the current NMA focuses on the primary prevention of overweight and obesity.

#### Types of interventions

RCTs with at least one nutritional component in the comparing study arms will be included. Eligible interventions include the entire school environment, including classrooms, cafeterias and canteens, vending machines, and tuck shops [40]. RCTs are included if one or more of the following nutrition components are present (the following categorization is based on reference [40]):

1. Nutrition education and literacy [45–48], e.g.:
  Children-oriented modules for the transfer of food and nutrition knowledge and skills
  Training of basic food preparation skills
  Measures for promoting food enjoyment and taste (e.g., tasting sessions)
  Parental involvement (e.g., parent information evenings, parent magazines/newsletters on healthy eating, nutrition workshops)
2. School-based food preparation [49]
  Joint preparation and consumption of (small) meals in school
  Parental involvement (e.g., parent-child cooking and meal preparations)
3. School garden programs [50]
4. Social marketing campaigns [45, 51, 52]
  Incentivizing healthy meal, snack, and beverage consumption (e.g., through raffles for sports items such as bicycles, basketball hoops, jump ropes, etc.)
  Increased advertising for healthy food and beverages
  Restrictions on the advertising of unhealthy foods and beverages
5. Nutrition-friendly school initiatives, e.g.:
  Improving school cafeteria food (e.g., implementing quality standards for lunch or breakfast [48, 53, 54]): (a) reduction of fat and/or salt intake [55, 56] and (b) reduction of sugar-enriched beverages [57, 58])
  Improving visibility and attractiveness of healthy foods (e.g., fruits and vegetables) in school cafeterias using environmental nudges [59]
  Directly providing healthy foods: (a) “5-a-day” fruit and vegetable initiatives [60–62] and (b) increased provision of school water fountains [63]
  Selling healthier foods, e.g., installing vending machines with healthier food [64]

RCTs comparing nutrition interventions with one another and/or a control (e.g., no intervention) will be included. RCT duration is not an exclusion criterion (similar to the Cochrane review on the HPS framework and students’ health, well-being, and school performance [35]).

The following interventions or measures will be excluded [40]:

1. Interventions focusing on health and safety questions
2. Food fortification programs for micronutrient (mineral and vitamin) deficiencies
3. Legislation on food and plant production or agricultural policy
4. BMI regulation (school report cards)
5. Alcohol regulations of any kind
6. Interventions focusing on eating disorders such as anorexia nervosa or bulimia

#### Types of outcome measures

Primary outcomes include the following anthropometric measures [35]: (a) risk (incidence/prevalence) of obesity or overweight (see Table 1 for definitions), (b) body weight change, (c) weight *Z*-score, (d) BMI or zBMI, (e) body fat, and (f) waist circumference. Secondary outcomes concern diet quality and include daily fruits and vegetable intake (separate and combined), daily fat intake, and daily intake of SSB [35].

**Table 1:** Definitions of overweight and obesity in school-aged children and adolescents

Organization	Definition of childhood overweight and obesity
World Health Organization	Children and adolescents aged 5–19 years (WHO 2007 Growth Reference [65]) ***BMI-for-age*** **Overweight:** BMI >1 standard deviation (SD) above WHO growth standard median (equivalent to BMI 25 kg/m^2^ at 19 years) **Obesity:** BMI >2 SDs above WHO growth standard median (equivalent to BMI 30 kg/m^2^ at 19 years)
Centers for Disease Control and Prevention (CDC)	Children and adolescents aged 2–20 years (*CDC 2000 Growth Charts* [66]*)* ***Sex- and age-specific BMI percentiles*** ***Overweight:*** *BMI ≥ 85th to 94th percentile* ***Obesity:*** *BMI ≥95th percentile*
International Obesity Task Force (IOTF)	Children and adolescents aged 2–18 years (IOTF 2000 Reference [67]) ***International sex- and age-specific BMI cut-off points***, linked to the adult BMI cut-off points 25 kg/m^2^ (overweight) and 30 kg/m^2^ (obesity)

Depending on whether anthropometric measures (e.g., body weight) were reported by children and parents (e.g., by telephone) or were objectively measured by study personnel [45], the data validity can be classified in different ways. Based on the judgment of the aforementioned Cochrane review, the anthropometric outcomes chosen can be classified as valid [35]. In general, food intake is measured using validated food frequency questionnaires (FFQ) [45], 24-h dietary recalls [68], or dietary records [60]. All available data on measuring instruments will be extracted from the respective primary studies. Based on the aforementioned Cochrane Review, outcome data will be initially extracted for outcomes that have been assessed up to immediately post-intervention (or closest to this time point, with a max. of 6 months post-intervention). Outcome data available and presented for >6 months after completion of the intervention are considered as post-intervention follow-up data and are also extracted.

#### Types of studies

We will include parallel RCTs and cluster RCTs with clusters at the school, district, or other geographical area level. As some nutrition interventions involve a holistic, total school approach (e.g., improving the quality of school cafeteria food), we will exclude studies with clusters only at the classroom level [35].

### Search strategy

Comprehensive systematic literature searches for relevant studies will be conducted in the following databases, without date or language restriction: PubMed, the Cochrane Library, Web of Science, Education Resources Information Center (ERIC), PsycINFO, CAB Abstracts, Campbell Library, Evidence for Policy and Practice Information and Co-ordinating Centre (EPPI-Centre) BiblioMap, Australian Education Index, Joanna Briggs Institute Evidence-Based Practice (JBI EBP) Database, and Practice-based Evidence in Nutrition (PEN) Database. In addition, reference lists from relevant studies assessed for eligibility will be screened and citations will be tracked to identify additional relevant articles. Furthermore, searches for ongoing or unpublished studies will be performed in ClinicalTrials.gov (https://clinicaltrials.gov), Current Controlled Trials, WHO International Clinical Trials Registry Platform (ICTRP), and the EPPI-Centre Trials Register of Promoting Health Interventions (TRoPHI). Table 2 shows an example search strategy for one of the eleven included electronic databases.

**Table 2:** Search strategy for the electronic database PubMed

Search	Query
#13	Search: **#3 OR #6 OR #10 OR #11** Filters: **Randomized Controlled Trial** Sort by: **Most Recent**
#12	Search: **#3 OR #6 OR #10 OR #11** Sort by: **Most Recent**
#11	Search: (“Child Nutrition Sciences”[MeSH Terms] AND (“School Health Services”[MeSH Terms] OR “Schools”[MeSH Terms] OR “school*”[all fields] OR “pre -school*”[Title/Abstract] OR “pre school*”[Title/Abstract] OR “kindergarten*”[Title/Abstract])) Sort by: **Most Recent**
#10	Search: **#7 AND #8 AND #9** Sort by: **Most Recent**
#9	Search: (“Schools”[Mesh] OR “Students”[Mesh] OR school* [tiab] OR preschool* [tiab] OR pre-school* [tiab] OR kindergarten* [tiab]) Sort by: **Most Recent**
#8	Search: (nutr* [tiab] OR diet* [tiab] OR food* [tiab] OR feed* [tiab] OR intake* [tiab] OR consum*[tiab] OR eating [tiab] OR habit* [tiab]) Sort by: **Most Recent**
#7	Search: (health* [tiab] AND (promot* [tiab] OR “policy” [tiab] OR “policies” [tiab] OR educat* [tiab] OR environment* [tiab] OR curricul* [tiab] OR intervention* [tiab] )) Sort by: **Most Recent**
#6	Search: **#4 OR #5** Sort by: **Most Recent**
#5	Search: “School based nutrition intervention*” [tiab] OR “school food polic*” [tiab] OR “school nutrition practice*” [tiab] OR “School Nutrition Polic*” [tiab] OR “school-based intervention*” [tiab] OR “school feeding program*”[tiab] OR “health-promoting school*” OR “school lunch*” [tiab] OR “cafeteria-based intervention*” [tiab] OR “school food service change*” [tiab] Sort by: **Most Recent**
#4	Search: (“nutrition polic*” [tiab] OR “nutrition promotion program*” [tiab] OR “Food Environment Polic*”[tiab] OR “food service intervention*” [tiab] OR “food service modificat*” [tiab] OR “Health behaviour intervention*” [tiab] OR “foodservice program*” [tiab] OR “nutrition curricul*” [tiab] OR “nutrition educat*” [tiab] OR “nutrition polic*” [tiab] OR “obesity prevention” [tiab] OR “Overweight prevention*” [tiab] OR “adiposity prevention*” [tiab] ) AND ( school* OR kindergarten) Sort by: **Most Recent**
#3	Search: **#1 AND #2** Sort by: **Most Recent**
#2	Search: **“Health Promotion” [MeSH] OR “Nutrition Policy” [MeSH] OR “Health Education”[Mesh] OR “Pediatric Obesity/prevention and control”[MeSH] OR “Obesity/prevention and control”[Mesh]** Sort by: **Most Recent**
#1	Search: (“School Health Services”[Mesh] OR “Schools” [Mesh] OR “school*”[tiab] OR “pre-school*”[tiab] OR “pre school*”[tiab] OR “kindergarten*”[tiab]) Sort by: **Most Recent**

### Study selection

All identified references will be imported into the Endnote reference manager [69] for removal of duplicates before they are uploaded to Covidence (http://www.covidence.org) for title, abstract, and full-text screening. Study selection will be performed in a two-step selection process. First, titles and abstracts of all identified references are independently screened by two reviewers (EN, JM) using the aforementioned eligibility criteria. After exclusion of non-eligible records, the full texts of potentially eligible references are retrieved in the second selection step and examined in more detail by the two reviewers. If an abstract is missing and the title of a reference appears to be potentially relevant, it will also proceed to full-text review. Study selection is based on the procedure recommended by Cochrane [70] for systematic reviews and is also carried out independently by at least two reviewers in both selection steps. Any disagreements between the reviewers during title, abstract, and full-text screening are resolved by discussion, with the involvement of a third reviewer (LS) if no agreement can be reached.

### Data extraction

Data extraction will also be performed independently by two reviewers (EN, JM). A data extraction sheet will be created and piloted a priori, after which all data is entered into an Excel spreadsheet. The following study characteristics will be extracted for each included study: first author (last name), year of publication, study design and duration, country, number of participants, participant characteristics (age, gender, BMI, proportion of overweight or obesity, socioeconomic status (SES), and migration background), description of setting or school type, number of schools, description of intervention and control arms, description of (possible) additional components of intervention and control arms, description of outcomes (including time of measurement, outcome assessor or measurer, dietary assessment instrument used (e.g., FFQ, 24h-recall, etc.), methods for anthropometry assessment (e.g., weight scale, stadiometer, measurement tape, etc.)), and funding source. We will extract risk ratios (RR) with 95% confidence intervals (CI) for dichotomous (binary) data and change from baseline values (change scores) with standard deviations for continuous data. Where available, we will extract change scores from an analysis of the covariance model (ANCOVA), followed by change scores. Missing change scores will be calculated from pre- and post-intervention using a correlation coefficient according to the formula provided by the *Cochrane Handbook* [70]. If studies considered the same endpoint but measured with different scales or instruments, we will first standardize results and then calculate standardized mean differences (SMDs). If reported data are separated for gender or age group, we will pool these data with a fixed effect MA. Study authors will be contacted in case of missing or unclear primary (study) data. If primary studies have not been adjusted for clustering, we will adjust the extracted data ourselves [71].

### Risk of bias assessment

Two reviewers (EN and JM) will independently assess the risk of bias of included studies using the revised Cochrane risk-of-bias tool for randomized trials (RoB 2) [72] and any disagreements will be resolved by consensus. The RoB 2 tool consists of five domains: bias arising from the randomization process, risk of bias due to deviations from the intended interventions, bias due to missing outcome data, bias in the measurement of the outcome, and bias in the selection of the reported results. The original RoB 2 tool will be used for parallel RCTs and a variant of it for cluster RCTs. The RoB 2 variant for cluster RCTs primarily focuses on cluster RCTs in which groups of individuals form the clusters [73, 74]. It should be noted that due to the pragmatic nature of cluster RCTs, in which intervention effects are usually examined under real-life conditions, this RoB 2 variant only takes into account the effect of assignment to the intervention (intention-to-treat) and not the effect of starting and adhering to the intervention as intended (per-protocol) [73, 74]. The RoB 2 tool for cluster RCTs consists of an additional domain, i.e., “bias arising from the timing of identification and recruitment of participants (at randomization)”, and the domain “bias due to deviations from the intended interventions” only concerns the effect of assignment to intervention. The overall risk of bias for a study will be judged as low, some concerns, or high risk in either version of the tool.

### Data synthesis

**Fig. 1:**
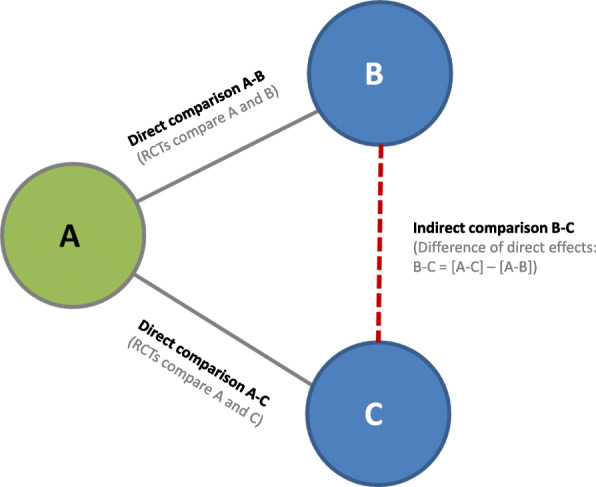
Determining the effect of an indirect comparison (B–C) in network meta-analysis

We will use NMA to synthesize all available data. While in a traditional pairwise MA only effects of individual studies can be combined for a single comparison at a time (e.g., nutrition education versus control), NMA allows the simultaneous comparison of multiple interventions while maintaining the internal randomization of individual trials [75]. For this purpose, NMA does not only include direct evidence, but also indirect evidence estimated from the available direct comparisons. This is possible, for example, when direct evidence is available for the comparison of intervention A (nutrition education) with B (nutrition-friendly school initiatives) and for the comparison of intervention A with C (control group). A subsequent indirect comparison of the relative effect of intervention B versus C can then be calculated by subtracting the effect of A–B from the effect of A–C (Fig. 1).

**Fig. 2:**
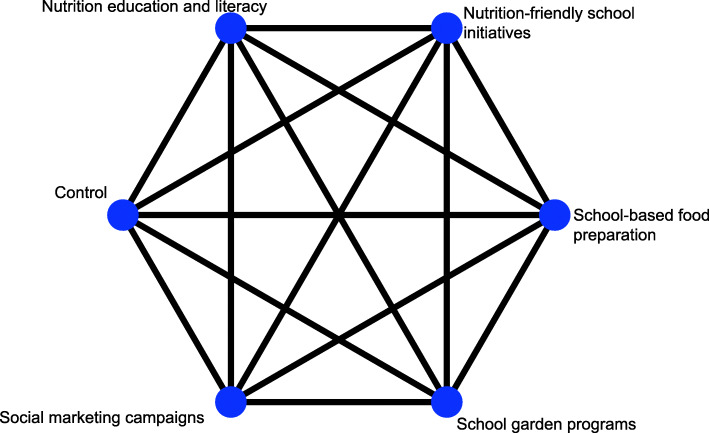
Hypothetical network plot of all possible pairwise comparisons of school-based nutrition interventions

This indirect effect is then pooled with the direct effects to produce the effect estimate of the NMA [75]. For each outcome, the available direct comparisons between different interventions and control groups are presented using a network plot [76] (Fig. 2). Nodes (circles) represent the different available intervention types and are proportional to the sample size of each intervention. Edges (lines) show the available direct comparisons between pairs of interventions and are displayed thicker when more studies are available for a comparison [77].

### Statistical analysis

Available direct comparisons between school-based nutrition interventions will be illustrated using a network plot for the following outcomes: overweight and obesity risk, body weight change, BMI or zBMI, body fat, or waist circumference, daily fruits and vegetable intake, daily fat intake, and daily intake of SSB. Afterwards, the direct and indirect treatment effects across the RCTs will be pooled, and effect estimates (RR, MD, SMD) for the outcome measures will be calculated.

Random effects pairwise meta-analysis will be performed for each outcome to estimate all possible pairwise relative effects in terms of change scores for the different school-based nutrition interventions where direct evidence is available from at least two studies. Between-study heterogeneity of results will then be explored using Cochranes *Q* test and *I*^2^ statistic, where an *I*^2^ >50% will be considered as representing substantial heterogeneity [78]. Forest plots will be created to display study specific and total effect estimates with corresponding 95 % CIs.

All available evidence will then be synthesized using NMA. We will perform NMAs in a contrast-based frequentist framework using the R package *netmeta*, Version 1.2-1 [79]. NMA results will be presented as summary effect estimates with 95% CIs using league tables, in which the NMA effects are compared with the pairwise effects. Interventions are then ranked according to the probability of being the most effective intervention for a certain outcome using *P*-scores. *P*-scores are a frequentist version of the Surface Under the Cumulative Ranking curve (SUCRA) [80, 81], with values between 0 and 1. A value of 1 indicates that an intervention always ranks best and a value of 0 means that an intervention always ranks worst [72].

In addition, we will use component NMA (CNMA) where possible and appropriate. CNMA allows breaking down the effects of complex interventions into their individual components [82]. Several CNMA models exist and may potentially be used to identify essential or “active” elements of complex interventions [82]. One of these models assumes that the effect of an intervention is the sum of the effects of its components (additive model). We will adopt the additive CNMA model to assess the contributions of the individual components of the different school-based nutrition intervention to their overall effect.

#### Assessment of transitivity

Prior to conducting any NMA, an assessment of the transitivity assumption is required. Briefly, the transitivity assumption indicates that studies comparing different groups of interventions are sufficiently similar to provide valid indirect conclusions. To assess transitivity, the distribution of possible effect modifiers across the available direct comparisons will be compared in advance. Transitivity applies if the distributions of all effect modifiers are comparable across the available direct comparisons [83]. To evaluate the assumption of transitivity [84], we will compare the similarity of the included populations and study settings in terms of age, gender, BMI, SES, and study length for the available direct comparisons.

#### Assessment of consistency

Intransitivity in the data can be the cause of statistical inconsistency. In the context of NMA, the term inconsistency refers to a statistical measure that describes the differences between direct and indirect evidence. To assess potential inconsistency, we will split the effect estimate for each comparison into the contribution of direct and indirect evidence to see whether they differ. In order to identify and display inconsistency in the network, we will create a net heat plot by applying a full treatment-design interaction model [83]. This model separates effects within and between different designs. A design is defined as the subset of treatments which are compared in a trial.

#### Secondary analyses and sensitivity analyses

If a sufficient number of studies are identified, we will conduct secondary analyses for intervention duration, gender, age of school students, SES, migration background [85], and geographical location (e.g., Germany). Sensitivity analyses will be performed by excluding RCTs rated as high RoB.

#### Dissemination bias

To evaluate dissemination bias, a comparison adjusted funnel plot [86] will be created for each direct pairwise comparison and Egger’s linear regression test for funnel plot asymmetry will be conducted to investigate small study effects [87].

### Grading of recommendations assessment, development, and evaluation (certainty of the evidence)

We will follow the GRADE approach to rate the certainty of evidence derived from NMA. For all outcomes, two authors independently (EN, LS) will rate the certainty of evidence in each of the direct, indirect, and network estimates [88]. Direct estimates will be evaluated with the following GRADE criteria: risk of bias, indirectness, inconsistency, and publication bias. As suggested recently by the GRADE working group, consideration of imprecision is not necessary when rating the direct and indirect estimates to inform the rating of NMA estimates [88]. We will use the certainty of direct estimates to inform indirect estimates (the lowest of the ratings of the two direct comparisons forming the most dominant first-order loop will be chosen), and eventually, we will rate them down in the presence of serious intransitivity (i.e., highly diverse population). We will compare respective ratings for direct and indirect estimates to address the certainty of network estimates (the one with higher certainty will be chosen) rated down if incoherence or imprecision will be present [88]. Overall, GRADE specifies four levels of certainty of evidence: high, moderate, low, and very low.

## Discussion

This systematic review and NMA will be the first to summarize and compare the effects of different nutrition intervention strategies for the primary prevention of overweight and obesity in school settings. By using both direct and indirect evidence, we will be able to not only compare but also rank interventions that have not been compared against each other yet. This may lead to novel and possibly important insights about the effects of different school-based nutrition intervention strategies. Our analysis will show which intervention strategies may be the most promising for the prevention of overweight and obesity in children and adolescents. We are confident that the current research project will significantly contribute to identifying gaps in the current evidence and help address issues related to the study design of existing studies on this topic. This may lead to new insights into target group specificity, in particular on intervention effects in different age and gender groups, which are also closely linked to contextual aspects. Furthermore, by using CNMA where appropriate, we may unravel intervention features that are essential for successful school-based overweight and obesity prevention strategies. Our findings may also provide useful insights to inform the design of new studies and nutrition interventions for different populations and settings. When designing new studies, all existing evidence and interventions for a specific research questions should ideally be considered. Up-to-date NMAs that include several different interventions that have not been directly compared with each other previously can help identify (new) promising interventions for specific health-related research questions through indirect comparisons and thus provide a solid basis for planning new studies. NMA has therefore been recommended for the optimal planning of the trial design as well as the estimation of the required sample size for new trials [89–92]. With regard to the planning and design of future school-based preventive measures, the current project will also provide interesting insights into whether and in what shape collaboration took place between (newly) implemented school nutrition intervention strategies and, for example, already existing school nutrition education classes and teachers. This might be of great importance and could lead to important recommendations, depending on the relevance of curricula-based nutrition education as a part of general education in primary and secondary schools in collaboration with nutrition intervention strategies in school settings. Overall, the findings of the present systematic review and NMA will be of great interest and value to both national as well as international public health authorities and policy makers when developing and implementing successful, evidence-based nutrition intervention strategies in school settings.

## Supplementary Information

**Additional file 1.** PRISMA-P checklist as applied to the current study protocol

## Data Availability

Not applicable

## Abbreviations

ANCOVA: Analysis of covariance
BMI: Body mass index
CDC: Centers for Disease Control and Prevention
CI: Confidence interval
CNMA: Component Network Meta-Analysis
CVD: Cardiovascular disease
EPPI-Centre: Evidence for Policy and Practice Information and Co-ordinating Centre
ERIC: Education Resources Information Center
FFQ: Food Frequency Questionnaires
GRADE: Grading of Recommendations Assessment, Development, and Evaluation
HPS: Health Promoting School
ICTRP: International Clinical Trials Registry Platform
IOTF: International Obesity Task Force
JBI EBP: Joanna Briggs Institute Evidence-Based Practice
PEN: Practice-based Evidence in Nutrition
PRISMA: Preferred Reporting Items for Systematic Reviews and Meta-Analyses
MA: Meta-analysis
MD: Mean difference
NMA: Network meta-analysis
RCT: Randomized controlled trials
RoB: Risk of bias
RR: Risk ratio
SD: Standard deviation
SES: Socioeconomic status
SMD: Standardized mean difference
SSB: Sugar-sweetened beverages
SUCRA: Surface under the cumulative ranking curve
TEI: Total energy intake
TRoPHI: Trials Register of Promoting Health Interventions
WHO: World Health Organization
zBMI: Body mass index z-score/standardized body mass index
